# Her-2/neu expression is a negative prognosticator in ovarian cancer cases that do not express the follicle stimulating hormone receptor (FSHR)

**DOI:** 10.1186/1757-2215-6-6

**Published:** 2013-01-22

**Authors:** Sabine Heublein, Thomas Vrekoussis, Doris Mayr, Klaus Friese, Miriam Lenhard, Udo Jeschke, Darius Dian

**Affiliations:** 1 Department of Obstetrics and Gynaecology - Campus Innenstadt, Ludwig-Maximilians-University of Munich, Maistrasse 11, 80337, Munich, Germany; 2 Department of Pathology, Ludwig-Maximilians-University of Munich, Thalkirchner Strasse 36, 80337, Munich, Germany; 3 Department of Obstetrics and Gynaecology - Campus Grosshadern, Ludwig-Maximilians-University of Munich, Marchioninistrasse 15, 81377, Munich, Germany

**Keywords:** Her-2/neu, Follicle stimulating hormone receptor, Ovarian cancer, Prognosis

## Abstract

**Background:**

Anti-Her-2 treatment is successfully administered to Her-2 overexpressing breast cancer patients and significantly implicates upon their survival. Building on these promising results, anti-Her-2 treatment protocols were tested as an option for epithelial ovarian cancer (EOC) as well. However Her-2 signalling is known to be modulated by G-protein coupled receptors (GPCR). Since a common GPCR in ovarian cancer is the FSH receptor (FSHR), we investigated the prognostic significance of Her-2 in patients that had been stratified according to their FSHR status.

**Findings:**

A total number of 153 EOC patients were included in this study. Her-2 positivity was assessed using a standard protocol. Intriguingly Her-2 turned out to be an independent prognostic marker for poor overall survival only in those patients that did not express FSHR. This did neither apply for the whole panel nor in case of FSHR co-expression.

**Conclusions:**

We thus conclude that Her-2 can be a negative prognosticator only in FSHR negative EOC cases. Hence by stratifying EOC patients according to their FSHR expression status, we introduce a diagnostic protocol to effectively select EOC patients that would most probably respond to anti-Her-2 treatment. This observation could be of clinical importance in terms of selecting the patient that would most likely benefit from anti-Her-2 treatment.

## Findings

### Background

The anti-Her-2 treatment is widely accepted in treating Her-2 expressing breast carcinoma cases, with a significant implication upon their survival [1]. These successful results triggered the optimistic hypothesis that anti-Her-2 treatment could be an option against any carcinoma expressing Her-2. The last decade epithelial ovarian cancer (EOC) has been reported as potentially Her-2 expressing, with a positivity ranging from 4.9% to 70.4% [2,3]. However its role in EOC survival is at least controversial. Despite this controversy and due to the significant impact of anti-Her-2 treatment on breast cancer, several groups have studied the role of trastuzumab or pertuzumab in Her-2 expressing EOC patients [4,5]. Interestingly, Her-2 has been reported as interacting with G-protein coupled receptors (GPCRs), both at a signalling and an extracellular level [6]. Of note is the fact that EOC has been reported as widely expressing follicle stimulating hormone receptor (FSHR) [7]. The FSHR expression has been found as a negative prognosticator in EOC patients [7]. Considering the interaction of Her-2 and FSH as possible, we have studied the effect of Her-2 in survival of EOC patients by stratifying them according to their FSHR status.

## Patients and methods

Formalin fixed paraffin embedded (FFPE) tissue of 153 EOC patients (mean age: 58.71 years) who had undergone surgery for EOC from 1990 to 2002 at our department were analysed. Histological characterization (serous: n = 108, mucinous: n = 12, endometrioid: n = 21, clear cell: n = 12) and WHO tumour grading (G1: n = 36, G2: n = 52, G3: n = 53) was performed by an experienced gynaecological pathologist (DM). Data on FIGO stage (I: n = 34, II: n = 9, III: n = 102, IV: n = 3) and survival (median: 3.41 years; deaths: n = 103) were available. Patients staged as FIGO II - IV received carboplatin/paclitaxel as an adjuvant chemotherapy. The study was carried out in compliance with the guidelines of the Helsinki Declaration (1975) and was approved by the Ethics Committee of the Ludwig-Maximilians-University of Munich.

FFPE ovarian cancer samples were stained using an FDA-approved Ventana PATHWAY anti-Her-2 (4B5) Rabbit monoclonal antibody (Roche, Mannheim, Germany) on a VENTANA®-Unit (Roche, Mannheim, Germany) and scored as recommended by the manufacturer as 3+, 2+, 1+ or 0 by two blinded independent observers including a gynaecological pathologist (DM) by consensus. Cases that were scored as 2+ underwent fluorescence in situ hybridisation (FISH) as described previously [8]. Immunohistochemical staining for the FSH receptor was assessed as already described by our group [7]. For survival analysis, samples expressing Her-2 at 1+, 2+ or 3+ on a protein level were scored as positive while median FSHR receptor expression (IRS = 3) was used as a cut off to define FSHR positive vs. negative tumours. Gamma/Spearman coefficients, uni- and multivariate survival analysis were employed for statistical analysis. Statistical significance for all tests was set as p < 0.05.

## Results

**Figure 1:**
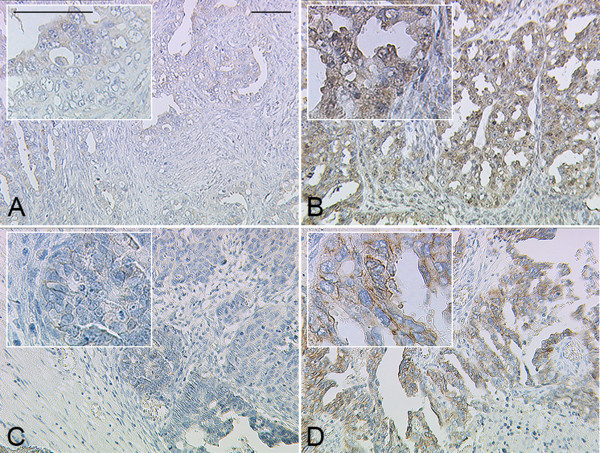
**Representative microphotographs of FSHR (A, B) and Her-2 (C, D) immunostaining are shown.** Patients were stratified according to their FSHR expression status (FSHR negative (**A**), FSHR positive (**B**)). The prognostic significance of Her-2 positivity (**D**) as compared to Her-2 non expressing (**C**) cases has been evaluated. Scale bars in (**A**) equal 100 μm and refer to A-D.

Immunohistochemistry (Figure 1) revealed 12 EOC samples (7.8%) as being Her-2 positive. A high Her-2 immunoreactivity was observed in just one case (3+, n = 1; 0.7%), while another 11 cases were moderately (2+, n = 2; 1.3%) or weakly (1+, n = 9; 5.9%) stained. None of the moderately (2+) expressing cases showed *ERBB2* amplification. Eighty two (53.6%) patients were evaluated as negative (IRS ≤ 3) for FSHR expression. Immunoreactivity of Her-2 and FSHR were positively correlated (rho = 0.21; p = 0.009), while Her-2 was not correlated to grade or FIGO stage.

**Figure 2:**
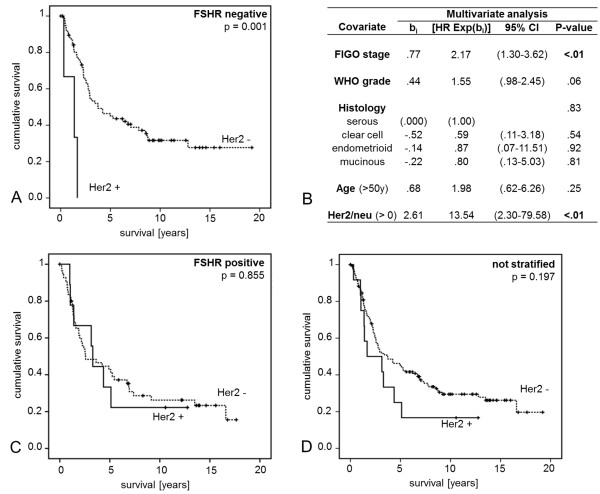
**Kaplan-Meier plots and multivariate Cox-Regression analysis in EOC patients.** Comparison of overall survival of Her-2 positive vs. negative cases (**D**) revealed no significant difference. However in FSHR negative cases (**A**) Her-2 positivity was strongly associated with shortened overall survival (p = 0.001) and turned out to be an independent prognosticator (**B**) in these patients. No link of Her-2 expression and prognosis could be observed in FSHR positive patients (**C**).

Kaplan-Meier univariate analysis revealed no different outcome of Her-2 expressing vs. non expressing cases though a trend for shortened overall survival was observed in case of Her-2 expression. However patients who did not express FSHR but were at the same time positive for Her-2 emerged to survive significantly shorter (p = 0.001) than those cases that did not express Her-2. Within the group of FSHR negative patients, Her-2 expression (p = 0.004) and FIGO stage (p = 0.003) turned out to be independent prognosticators (Figure 2).

## Discussion

The role of Her-2 in EOC is still unclear. Several studies support a potential role upon EOC survival [9] while others consider it as not being related to prognosis [8]. This can be mainly attributed to the fact that up to-date there is no consensus about the evaluation protocol applied for Her-2 overexpression in EOC. The situation became even more complex due to the fact that different antibodies are used. It can thus be concluded that regarding Her-2 in EOC there is a far more imperative need at least for a consensus in the way Her-2 overexpression will be evaluated.

The existing reports upon efficacy of anti-Her-2 treatment in ovarian cancer patients are few and not encouraging, mainly because the response rate was rather low [4,5]. Such finding though can be explained possibly by the hypothesis that other molecular pathways interfere with the EGFR/Her-2 signalling [6,10], potentially minimizing Her-2 impact on ovarian cancer cell proliferation and ultimately to the Her-2 effect on disease progression and prognosis.

Herein we hypothesized that such interference could involve the FSHR and its downstream signalling. Although FSHR-expressing EOC patients have been shown to be of worse prognosis compared to FSHR non-expressing EOC patients [7], an effective selective anti-FSHR intervention has not been published so far. Several clinical trials using anti-gonadotropin receptor (GnR) agents have failed to demonstrate a significant improvement in EOC patient survival [11,12], most possibly due to the fact that such treatments target simultaneously the luteinizing hormone receptor (LHR) as well, a receptor being a positive prognosticator for EOC survival. Thus, anti-GnR failure can be attributed to the combined cancelling of a negative and a positive prognosticator.

Despite the failure of anti-GnR treatment, the idea of using FSHR - a negative prognosticator for EOC - as a stratifying factor in order to evaluate Her-2 impact on EOC survival, is still appealing. Most possibly FSHR signalling by interfering with Her-2 could abrogate Her-2 signalling and thus its effect on survival. The results presented herein verify this hypothesis. Only in FSHR negative EOC cases does the Her-2 expression present a significant negative impact on patient survival. Of course it could be argued that the sample size of Her-2 positive cases presented herein is rather small. We would agree on such comment, highlighting at the same time though the fact that Her-2 expression in EOC is also restricted. Additionally, by dissecting the Her-2 expressing cases into FSHR positive and negative, we provide an explanation for the restricted impact of anti-Her-2 treatment in EOC. It can be hypothesized that it is likely for EOC patients with the specific phenotype (FSHR negative/Her-2 positive) to benefit from anti-Her-2 treatment. Since, though such patients consist a very small proportion of EOC patients (in our series 3 out of 153), a multicentre clinical study would be necessary to obtain an adequate sample to prove in a statistically significant way whether our current findings and hypothesis are of clinical importance.

## Conclusions

In the current report it is demonstrated that Her-2 can be a negative prognosticator only in FSHR negative EOC cases. This observation could be of clinical importance in terms of selecting the patient that would probably benefit from anti-Her-2 treatment. Hence by stratifying EOC patients according to their FSHR expression status, we introduce a diagnostic protocol to effectively select EOC patients that would most probably respond to anti-Her-2 treatment. This observation could be of clinical importance in terms of selecting the patient that would most likely benefit from anti-Her-2 treatment. However due to the small incidence of the FSHR positive/Her-2 negative phenotype in EOC a multicentre study is necessary to verify our result and - as a second step - to assess anti-Her-2 treatment efficacy.

## Abbreviations

Her-2: Receptor tyrosine-protein kinase erbB-2; FSHR: Follicle stimulating hormone receptor; LHCGR: Luteinizing hormone receptor; EGFR: Epidermal growth factor receptor; IRS: Immuno reactive score; EOC: Epithelial ovarian cancer; FFPE: Formalin fixed paraffin embedded; FISH: Fluorescence in situ hybridisation; FDA: Food and drug administration; FIGO: International Federation of Gynecology and Obstetrics.

## Competing interests

The authors declare no conflict of interest.

## Authors’ contributions

SH significantly contributed to acquisition and evaluation of the immunohistochemical data and performed the statistical analysis. SH and TV drafted the manuscript. DM analysed the tissue samples and the immunohistochemical staining as a gynaecological pathologist. ML and UJ initiated the study. Design and coordination of the study was performed by KF, UJ and DD. All authors read and approved the final version of the manuscript.

## Authors’ information

Udo Jeschke and Darius Dian share senior authorship.
